# Obesity, food insecurity, and depression among females

**DOI:** 10.1186/s13690-020-00463-6

**Published:** 2020-09-17

**Authors:** Manik Ahuja, Thiveya Sathiyaseelan, Rajvi J. Wani, Praveen Fernandopulle

**Affiliations:** 1 College of Public Health, East Tennessee State University, 41B Lamb Hall, Johnson City, TN 37614 USA; 2 Aureus School of Medicine, Oranjestad, Aruba; 3 College of Public Health, University of Nebraska Medical Center, Omaha, NE USA; 4 Psychiatry and Behavioral Sciences Department, East Tennessee State University, Johnson City, TN USA

**Keywords:** Food insecurity and mental health, Obesity and depression, Food insecurity and obesity among women, Obesity and mental health disorders

## Abstract

**Background:**

Nutritional psychiatry is an emerging field of research and it is currently exploring the impact of nutrition and obesity on brain function and mental illness. Prior studies links between obesity, nutrition and depression among women. However, less is known how food insecurity may moderate that relationship.

**Methods:**

Data were employed from the Collaborative Psychiatric Epidemiology Surveys (CPES), 2001–2003. Two logistic regression models were Logistic regression was used to determine the association between obesity, gender, food insecurity, and past year Major Depressive Disorder (MDD). We then stratified by gender, and tested the association between obesity and past year MDD, and if food insecurity moderated the association.

**Results:**

Obesity was associated with an increased risk for past year Major Depressive Disorder (MDD) among females (AOR = 1.35; 95% CI 1.17–1.55) and was not associated among males (AOR = 1.07; 95% CI, 0.86–1.32). Women who reported that reported both obesity and food insecurity reported higher odds of past year MDD episode (AOR = 3.16; 95% CI, 2.36–4.21, than women who did not report food insecurity (AOR = 1.08; 95% CI, 1.02–1.38).

**Conclusion:**

With rising rates of mental health problems, females should be closely monitored to understand how poor diets, food insecurity, and obesity play a role in mental health outcomes. It is recommended that clinicians and treatment providers consider the patient’s diet and access to nutritious foods when conducting their assessment.

## Background

Nutritional psychiatry is an emerging field of research and it is currently exploring the impact of nutrition on brain function and mental illness. Nutrition has been vastly overlooked as a contributor to mental health problems [1]. The prevalence of obesity in the adult population is a global health challenge [2], and is associated with adverse outcomes including chronic disease and mortality. Obesity is defined as Body Mass Index (BMI) of 30 or more, and known to highly prevalent among individuals who consume inexpensive, calorie-dense foods and engage in lower levels of physical activity [3–6].

Several studies have found associations between obesity and mental health problems [7–9]. Simon and his colleagues (2006) concluded that obesity is associated with approximately 25% increase in odds of mood and anxiety disorders [10]. A large cross-sectional study conducted by McMartin et al. [11] reported consistent inverse relationships between fruit and vegetable intake and major depressive disorder (MDD) [12]. The associations between dietary patterns and depression remains significant and strong epidemiological evidence suggests that poor diet can have a negative effect on mental health disorders. Implementing changes to the diet is gaining popularity in life sciences as it provides structural, functional, and biochemical roles of macro- and micronutrients in mental well-being [13].

Studies have revealed positive associations between obesity and depressive symptoms among women and either negative or no associations among men [8, 10, 14, 15]. Studies show there are significant gender disparities, as women are nearly twice as likely as men to suffer from mental illness [16, 17]. According to data from the National Health and Nutrition Examination Survey (NHANES), the rate of obesity for adult women is over 40% in the U.S. [18], and is expected to continue to rise over the next 30 years [19], placing even more women at risk. In addition, obesity and depression both carry an increased risk for other diseases such as cardiovascular disease [20]. There are still more gaps to fill when it comes to understanding obesity and mental health among women. The dietary intake in the female population in conjunction with the biochemical measurements of diet can aid in distinguishing the cause of MDD, whether it is caused by reduced intake, altered metabolism or enzymatic function [21].

For many there is limited access to nutritious food as it can be costly, therefore, individuals often consume poor diets. As food insecurity impacts 1 in 7 people in the United States and it can also be a contributor to the rising obesity rate [22]. Prior studies have found associations between food insecurity and poor mental health among females [23, 24]. To date, no studies have investigated how food insecurity moderates the effects of obesity and depression particularly among women. To current study addresses these gaps using a nationally representative, ethnically diverse sample from the United States.

## Methods

### Data

The current study used secondary data from the National Co-Morbidity Survey Replication (NCS-R), National Survey of American Life (NSAL), and National Latino and Asian American Study (NLAAS), as these were presented in the Collaborative Psychiatric Epidemiology Surveys (CPES), 2001–2003. CPES was designed to collect representative samples of majority and minority adult populations in the U.S., with oversampling of race/ethnic minorities [25]. The study employed probability sampling techniques to identify 252 geographic areas across the U.S., where adults were selected from each eligible household [25]. For specific study design see [26–28]. The final sample included 20,013 respondents (aged > = 18), who self-identified as female (*n* = 11,463; 57.3%), male (*n* = 8550; 42.7%), non-Hispanic white (*n* = 7.587; 37.9%), black (*n* = 6.281; 31.1%), Latino (*n* = 3.620; 18.1%), and Asian (*n* = 2.284; 11.4%). The CPES data were obtained from the Inter-university Consortium for Political and Social Research (Ann Arbor, MI). All procedures that involved human subjects, including consent from all participants 18 years of age and older, were approved by the Institutional Review Board at the University of Michigan [29].

## Methods

### Data

The current study used data from the National Co-Morbidity Survey Replication (NCS-R), National Survey of American Life (NSAL), and National Latino and Asian American Study (NLAAS), as these were presented in the Collaborative Psychiatric Epidemiology Surveys (CPES), 2001–2003. The final sample included 20,013 respondents (with complete information on all variables in the analyses) who self-identified as female (*n* = 11,463; 57.3%) and male (*n* = 8550; 42.7%), non-Hispanic white (*n* = 7.587; 37.9%), Black (*n* = 6.281; 31.1%), Latino (*n* = 3.620; 18.1%), and Asian (*n* = 2.284; 11.4%).

### Measures

The primary outcome of interest in the current study is report of past year Major Depressive Disorder (MDD) episode. Past year episode of MDD was assessed based on lifetime report of MDD, as measured by the diagnostic interview of World Mental Health Initiative version of the composite International Diagnostic Interviews, which is based on DSM-IV criteria (APA [30]).

Obesity was coded binary, with participants who reported a BMI level ≥ 30 kg/meter squared as 1, and BMI level < 30 as 0. BMI was determined participant from self-reports of height and weight. This coding of this measure is consistent with prior studies on obesity and depression (Gavin et al. [31]). Respondents were classified as either obese (BMI ≥30 kg/meter squared [kg/m^2^]) or non-obese (BMI 18.5–29.9 kg/m^2^) both for consistency with previous research and a lack of evidence suggesting that overweight status is statistically associated with depression. Food insecurity was based on the following question ‘In past yr-how often not enough money to buy food?’, and coded binary with 1 representing any report of food insecurity in the past year. This variable ‘food insecurity’ was selected based on its definition of having inconsistent access to adequate food because of limited financial and other resources [32], and has been applied in prior studies [33–35].

### Covariates

We adjusted for income, education, age, and race. Income was coded as 1 representing low income of <$30,000, and 0 representing income > = $30,000. Education was coded as <high school as 1, and 0 represents graduating high school or more than high school education. Race was coded based on self-report of race, and dummy coded into 4 variables including Black, Latino, Asian, with White representing the reference group.

### Data analysis

Two separate logistic regression models were run. In the first analysis, logistic regression was used to assess whether the obesity-depression association varied by gender. Interaction terms between obesity and gender were created and re-parameterized as ‘obesity and male’ and ‘obesity and female’. In the second analysis, we stratified by gender to assess whether there was a moderating effect of food insecurity. Data were re-parameterized as report of ‘obesity and food insecurity’, and ‘obesity and no report of food insecurity’, to assess whether food insecurity moderated the relationship between obesity and past year depression among females.

### Results

Table 1 shows descriptive statistics for the sample. Overall, (*n* = 4999); 25.1% of participants reported obesity at BMI > =30, overall (*n* = 1543; 7.7%) participants reported past year MDD, and (*n* = 1942; 9.7%) reported past year food insecurity.

**Table 1:** Sample characteristics and prevalence of BMI, food insecurity, and major depressive disorder (*N* = 20,013)

	N (%)
Sex	
Female	11,463 (57.3)
Male	8550 (42.7)
Race	
White	7587 (37.9)
Black	6238 (31.1)
Latino	3620 (18.1)
Asian	2284 (11.4)
Education^a^	
< High school	4112 (20.6)
Graduated High School	5919 (29.6)
Some College	5263 (26.3)
Graduated College	4719 (23.6)
Household income	
< $30,000	10,513 (52.5)
$30,000–$74,999	5944 (29.7)
> =$75,000	3556 (17.8)
BMI	
< 18.5: Underweight	582 (2.9)
18–5-24.9: Healthy weight	7285 (36.4)
25.0–29.9: Overweight	6576 (32.9)
> =30.0 Obesity	4999 (25.1)
Food insecurity	1942 (9.7)
Major depressive disorder^b^	1942 (9.7)
Lifetime episode	3241 (16.2)
Past year episode	1543 (7.7)

*N* Number, *BMI* Body Mass Index

^a^Highest level of education

^b^DSM-IV

In Table 2, we examined the association between obesity and 12-month MDD. In our fully adjusted model examining the main effects, obesity (AOR = 1.26; 95% CI 1.12–1.41), male gender (AOR = 0.57; 95% CI 0.51–0.64), and food insecurity (AOR = 3.29; 95% CI 2.82–3.83) were associated with past year MDD. We tested for interactions for gender and obesity, and found that females who reported obesity (AOR = 1.35; 95% CI 1.17–1.55) reported higher odds of past year MDD than males (AOR = 1.07; 95% CI, 0.86–1.32).

**Table 2:** Results of logistic regression analyses predicting past year major depressive disorder episode

	AOR (95% CI)
*Main effects*	
Non-Hispanic White	Ref
Black	0.66 (0.58–0.75)*
Latino	0.61 (0.52–0.71)*
Asian	0.36 (0.29–0.44)*
Male gender	0.57 (0.51–0.64)*
Female gender	Ref
Age	0.98 (0.98–0.99)*
Education: < High school	1.34 (1.17–1.53)*
Income: <$30,000 per year	0.84 (0.75–0.93)*
Food insecurity	3.29 (2.82–3.83)*
Obesity	1.26 (1.12–1.41)*
*Interactions*	
Obesity*female	1.35 (1.17–1.55)*
Obesity*male	1.07 (0.86–1.32)

*AOR* Adjusted odds Ratio, *CI* Confidence Interval, *Ref* Reference category

*significant (*p* < .05)

In Table 3, we used logistic regression to examine the effect of food insecurity as a moderating variable with obesity and MDD among females. We found that obese participants that reported food insecurity (AOR = 3.16; 95% CI, 2.36–4.21) had significant higher odds than participants who did not report food insecurity (AOR = 1.08; 95% CI, 1.02–1.38).

**Table 3:** The association of obesity, food insecurity and past year major depressive disorder (MDD) episode among females

Variable	AOR (95% CI)
Non-Hispanic White	Ref
Black	0.63 (0.54--0.73)*
Latino	0.78 (0.75–1.00)
Asian	0.40 (0.30–0.53)*
Age	0.98 (0.97–0.98)*
Education: < High school	1.44 (1.17–1.78)*
Income: <$30,000 per year	0.88 (0.77–1.01)
Obesity*food insecurity	3.16 (2.36–4.21)*
Obesity*no report of food insecurity	1.08 (1.02–1.38)*

*AOR* Adjusted odds ratio, *CI* Confidence Interval, *Ref* Reference category

*significant (*p* < .05)

## Discussion

This study reveals the association between obesity and depression and the moderating role of food insecurity that exists among women using a nationally representative sample from the United States. The study found an association between obesity and past year episode of MDD among women, but not men, which is consistent with prior research [8, 36, 37]. Depression may be associated with obesity due to factors such as negative body image, and low self-esteem [38, 39], which is more prevalent among women [40]. There are many potential factors that contribute to higher rates of depression among women, and both obesity and food insecurity are important factors to consider.

We looked at differences within women and found that women with high BMI levels (> = 30) and who reported food insecurity were at a significantly higher risk for depression than women who did not report food insecurity. Food insecurity is an important factor of health and nutrition outcomes [41]. Food insecurity may refer to individuals who are micronutrient deficient, or undernourished [42]. Food insecurity is also the uncertainty of acquiring nutritious food in a safe and socially acceptable manner Food insecurity has been found to be associated with obesity and unhealthy dietary patterns, both of which have negative health outcomes [43].

Given the increasing number of female-headed households, and the lower wages of women compared to men, women need special consideration in discussions of food insecurity and their effect on health [44]. Food insecurity is known to disproportionally affect women. A cross-sectional study using data from the nationally representative *Continuing Survey of Food Intakes by Individuals*, found that food insecurity was associated to overweight status in women in the United States, but not men [45]. In another study using data from a nationally representative sample, Hanson et al. [43] found that low food security was associated with being obese among women [46].

The findings of the study reveal that there are severe implications associated with food insecurity, particularly with mental health among women. Targeted interventions to increase awareness about safe, nutritious foods among women should be considered. Nutrition education plays an important role in food security [43]. For example, one study found that nutrition education was shown to improve the food security of low-income individuals who participated in the *Expanded Food and Nutrition Education Programs* in two states [46]. Food-insecure women have lower intakes of healthy foods such as fruits and vegetables and other nutrients compared with food-secure women [47, 48], It is important that women be given special consideration, and policy measures to improve access to safe, nutritious foods for women should be considered. These include public assistance programs, and other subsidies offered to women, which may prevent or reduce their risk of feeling food insecure. Given the current economic state, and the COVID-19 pandemic, women as well as children could feel the majority of the negative effects of food insecurity [49].

The results from this study should not be interpreted without considering the limitations. Firstly, the cause-effect relationships cannot be established in this study, given cross-sectional data were used. Second, was that surveys from non-English speaking Latinos and Asians were not collected in the NCS-R data. Third, depression diagnosis was based on DSM-IV criteria, as DSM-5 is currently implemented. There have been changes to diagnostic criteria, including the dropping of the Global Assessment of Functioning (GAF) scale, which was used in DSM-IV. This may pose a challenge among clinicians, who are using DSM-5 diagnosis to treat depression. However, the changes from DSM-IV to DSM-5 are not likely to significantly impact prevalence rates, and not affect the characteristics of diagnoses [50]. Fourth, BMI was calculated using self-reported height and weight by each participant. Prior studies have found that it is common for individuals to inaccurately report their height and weight, that include both over and underreporting measurements [51]. There are known discrepancies in these measurements when they are taken by a trained professional [51].

## Conclusion

The impact of nutrition and obesity on mental health and overall fitness is being reported and documented. To fill the gap in the current knowledge and literature base, this study demonstrates an association between obesity and mental health outcomes by gender while evaluating the effect of food insecurity. It is recommended that clinicians allocate time in discussing vital nutrients when formulating a treatment plan, as diet can augment the medications used to treat and prevent various chronic diseases. It is also vital for health providers to also consider food insecurity in their assessment as it makes it difficult for patients to adhere to a diet that supports their diagnosis.

## Data Availability

The datasets used and/or analysed during the current study are available from the corresponding author on reasonable request.

## Abbreviations

BMI: Body mass index
DHA: Docosahexaenoic acid
MDD: Major Depressive Disorder
PUFA: Polyunsaturated fatty acids
WHO: World Health Organization
